# Proximity Plus Pollution: Understanding Factors in Asthma among Children Living near Major Roadways

**DOI:** 10.1289/ehp.120-a436b

**Published:** 2012-11-01

**Authors:** Julia R. Barrett

**Affiliations:** Julia R. Barrett, MS, ELS, a Madison, WI–based science writer and editor, has written for *EHP* since 1996. She is a member of the National Association of Science Writers and the Board of Editors in the Life Sciences.

Compact urban development would reduce urban sprawl, leading to shorter driving distances and ultimately less regional air pollution. But it would also mean greater housing density in a given area, potentially increasing the number of residences near major roadways. Given that exposure to traffic emissions near roadways is strongly associated with asthma and related symptoms in children, a new study focuses on how air pollution reduction paired with changes in the proportion of children living near major roadways might affect overall rates of asthma-related outcomes within an urban population [*EHP* 120(11):1619–1626; Perez et al.].

Traditionally, studies of air pollution and asthma have focused on acute effects—that is, exacerbation of asthma symptoms caused by traffic-related exposure. This work distinguishes between direct effects of regional pollution on asthma symptoms and longer-term effects of living near a roadway on the development of asthma.

Previously collected data were used to estimate the prevalence of asthma and the occurrence of asthma-related outcomes (e.g., bronchitis episodes) in Los Angeles County in 2007. Los Angeles County roadway locations were paired with census and community data to determine the proportion of children living within 75 m of a major roadway. Monitoring stations provided annual average daily concentrations of sample regional traffic-related and secondary pollutants.

**Figure:**
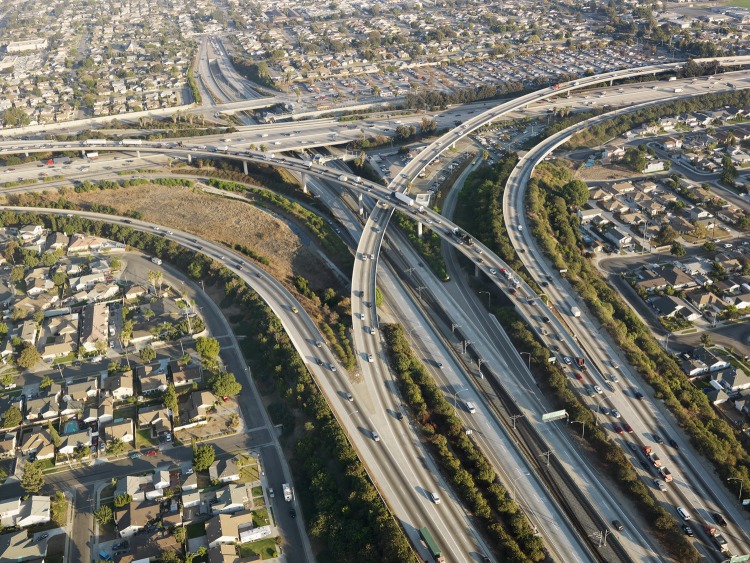
Near-roadway housing in Los Angeles, CA © Ron Chapple/Corbis

Nearly 18% of Los Angeles County children lived within 75 m of a major roadway. The authors estimated that approximately 27,100 asthma cases (8% of the total reported) could be at least partly attributed to living near a major roadway, whereas the combined effects of traffic proximity and regional nitrogen dioxide explained an estimated 70,200 episodes of bronchitis among children with asthma. If regional pollution were reduced by 20% but 3.6% more children (based on total county population) lived near a major roadway, an estimated 5,900 more cases of asthma would occur; if 3.6% fewer children lived by a major roadway with the same reduction in pollution, the estimated number of cases would drop by 5,900.

Reducing regional pollution by 20% would result in 19,900 fewer episodes of bronchitis, assuming 3.6% fewer children lived close to a major roadway. There would be 15,580 fewer episodes if the 20% decrease in regional pollution was accompanied by a 3.6% increase in proportion of children living near major roadways.

The results underscore the importance of considering near-roadway pollution exposures in urban planning, especially since these exposures may also contribute to atherosclerotic heart disease, chronic obstructive pulmonary disease, lung cancer, and adverse childhood neurodevelopmental outcomes. They conclude that compact urban design should be accompanied by strategies to mitigate exposure to near-roadway pollution.

